# Unveiling the dimension of regional disparities: Assessing the disruption of immunisation services by COVID-19 in Bangladesh

**DOI:** 10.7189/jogh.14.05028

**Published:** 2024-10-25

**Authors:** Ema Akter, Abu Sayeed, Abu Bakkar Siddique, Bibek Ahamed, Ridwana Maher Manna, Lubna Hossain, KM Tanvir, Md Ariful Islam Sanim, Md Hafizur Rahman, Srizan Chowdhury, Tasnu Ara, Md Alamgir Hossain, M Sabbir Haider, Sabrina Jabeen, Shafiqul Ameen, Mohammad Sohel Shomik, Anisuddin Ahmed, Luis Huicho, Alicia Matijasevich, Abdoulaye Maiga, Ahmed Ehsanur Rahman, Nadia Akseer, Shams El Arifeen, Aniqa Tasnim Hossain, Agbessi Amouzou

**Affiliations:** 1 Maternal and Child Health Division, International Centre for Diarrhoeal Disease Research, Bangladesh; 2 Institute of Statistical Research & Training (ISRT), Dhaka University, Bangladesh; 3 Institute of Epidemiology, Disease Control and Research (IEDCR), Bangladesh; 4 Nutrition and Clinical Services Division, International Centre for Diarrhoeal Disease Research, Bangladesh; 5 Centro de Investigación en Salud Materna e Infantil, Centro de Investigación para el Desarrollo Integral y Sostenible and Facultad de Medicina, Universidad Peruana Cayetano Heredia, Lima, Peru; 6 Departamento de Medicina Preventiva, Faculdade de Medicina FMUSP, Universidade de São Paulo, São Paulo, Brasil; 7 Department of International Health, Johns Hopkins University Bloomberg School of Public Health, Baltimore, Maryland, USA

## Abstract

**Background:**

The coronavirus disease 2019 (COVID-19) pandemic disrupted essential health care services worldwide, including those related to immunisation. National data from Bangladesh shows that child immunisation may have been adversely affected by the pandemic but regional evidence is limited. We therefore aimed to explore the regional differences in the indirect effects of COVID-19 on child immunisation in Bangladesh.

**Methods:**

We extracted data from the District Health Information Software (DHIS2) spanning the period from January 2017 to December 2021. We examined three essential immunisation indicators: *Bacille Calmette-Guérin* (BCG), pentavalent third dose, and measles vaccinations. We examined both the yearly and monthly trends to explore fluctuations in the number of immunisations to pinpoint specific periods of service utilisation regression. Segmented regression with Poisson distribution was implemented given the count-based outcome. We reported incidence rate ratios (IRRs) with 95% confidence intervals (CIs) in different regions in 2020 and 2021 compared to the reference period (2017–19).

**Results:**

We initially observed a notable decline in vaccine administration in April 2020 compared to the pre-pandemic period of 2017–19 with a drop of approximately 53% for BCG vaccines, 55% for pentavalent third doses, and 51% for measles vaccines followed by May 2020. The second half of 2020 saw an increase in vaccination numbers. There were noticeable regional disparities, with Sylhet (IRR = 0.75; 95% CI = 0.67–0.84 for pentavalent administration, IRR = 0.79; 95% CI = 0.71–0.88 for measles administration) and Chattogram (IRR = 0.77; 95% CI = 0.72–0.83 for BCG administration) experiencing the most significant reductions in 2020. In April 2020, Dhaka also experienced the largest decline of 67% in measles vaccination. In 2021, most divisions experienced a rebound in BCG and pentavalent administration, exceeding 2019 levels, except for Chittagong, where numbers continued to decline, falling below the 2019 figure.

**Conclusions:**

Our findings highlight the impact of the COVID-19 pandemic on childhood immunisation across regions in Bangladesh. Sylhet, Chattogram, and Dhaka divisions experienced the most significant reductions in immunisation services during 2020. This underscores the importance of targeted interventions and regional strategies to mitigate the indirect effects of future challenges on essential health care services, particularly childhood immunisation, in Bangladesh.

The World Health Organization (WHO) declared coronavirus disease 2019 (COVID-19) a pandemic on 11 March 2020, citing the emergence of cases in more than 110 countries and territories and the continued risk of global spread at that time [1]. While the establishment of social lockdowns, quarantines, physical distancing measures, and other interventions were crucial to mitigating the effects of the pandemic, they unintentionally disrupted access to essential health care services [2,3].

Immunisation has proven to be one of the most successful and cost-effective public health interventions to date [4]. It is estimated that a USD 1 investment in child immunisation results in a return of USD 16 due to the avoidance of illness, or up to USD 44 when the value of living longer and healthier lives is considered [5]. It is therefore worrying that the COVID-19 pandemic has disrupted routine child immunisation services globally and threatened several decades of progress in the control of vaccine-preventable diseases (VPDs) [6]. According to data collected by the WHO, United Nations International Children's Emergency Fund (UNICEF), Gavi, and the Sabin Vaccine Institute, routine immunisation services were severely impacted in at least 68 countries, affecting an estimated 80 million children under the age of one [7].

A scoping review on the effects of COVID-19 on other essential health services found that most research in this area focussed on high-income countries [8]. Several studies have found that COVID-19 specifically impacted health care utilisation in low- and middle-income countries (LMICs) [9–14]. However, not all health services, facilities, or geographic areas were affected in the same way. According to a study conducted in Bangladesh and Uganda, COVID-19 had a significant impact on all services related to maternal and child health, particularly in the early months of the pandemic [9]. Ayele et al. [14] found that essential health care services in nine selected health centres in Ethiopia were either completely or partially disrupted during the pandemic. Another study estimated that maternal and child services declined by 2.6–4.6% on average due to COVID-19 across the 18 LMICs [12]. Hategeka et al. [10], meanwhile, reported that COVID-19 largely had no effect on vaccinations in Kinshasa. Shapira et al. [11], in turn, found a cumulative shortage of 5 149 491 outpatient consultations and 328 961 third-dose pentavalent vaccinations in eight sub-Saharan African countries during the first five months of the pandemic. Each of these studies improved our knowledge of the degree of COVID-19 disruptions to health care systems. It is worth noting, however, that the methodological approaches, services analysed, and timeframes covered differed between these studies. Moreover, the effects of COVID-19 on the use of child immunisation services in different geographical regions of Bangladesh have not been explored, with a prior study only reporting changes at the national level [9]. The different regions in Bangladesh, each with distinct geographical, economic, and health care infrastructures, are likely to experience varied challenges in the face of public health crises. This diversity may have lead to different patterns of disruptions in immunisation across regions in the country. Studying these variations is essential for understanding the resilience of health care systems across different regions.

We aimed to explore the regional differences across the eight divisions of Bangladesh by assessing the indirect effects of COVID-19 on administration of immunisation through the use of routine health information data. Documenting the utilisation of immunisation at regional level is vital as it helps policymakers make decisions based on local conditions.

## METHODS

### Study settings

**Figure 1:**
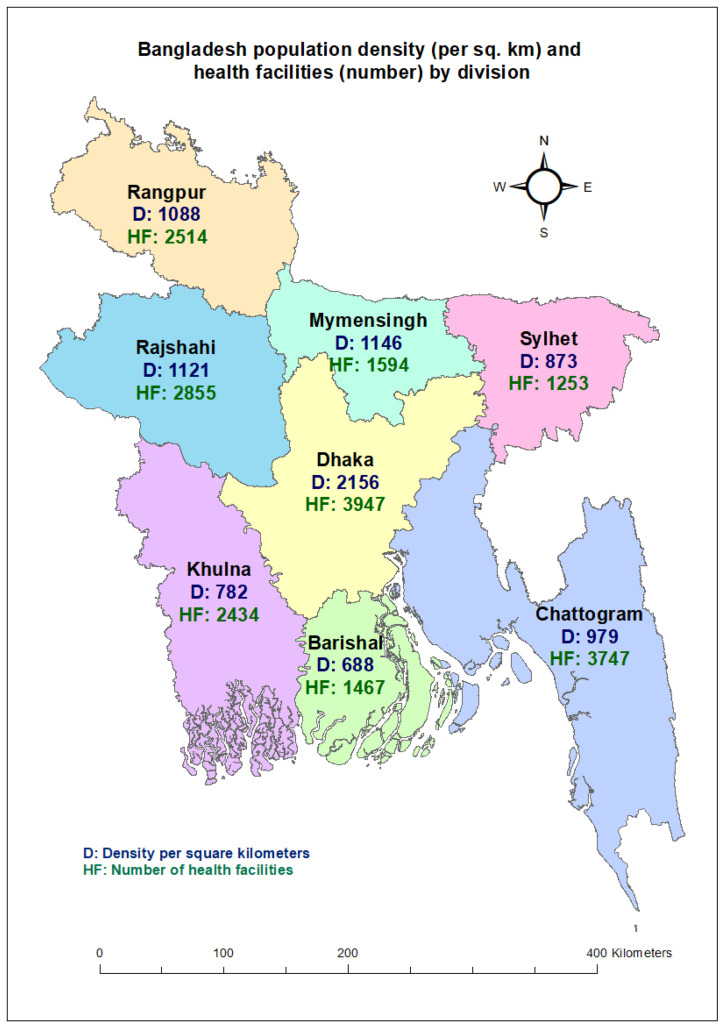
Population density and number of health facilities by division.

We used data on service utilisation in health care facilities across Bangladesh, a South Asian country with a diverse population. As per the Population and Housing Census 2022, Bangladesh has a population of approximately 165 million, making it one of the most densely populated countries globally, with a density of 1119 people per square kilometre [15]. Administratively, it is divided into eight divisions – Barishal, Chattogram, Dhaka, Khulna, Rajshahi, Rangpur, Mymensingh, and Sylhet – among which Dhaka has the highest population density of 2156 people per square kilometre (**Figure 1**). The health service in Bangladesh is supervised by the Ministry of Health and Family Welfare, which records a total of 19 811 registered health facilities under its purview [16].

### Data source

The WHO recommends the District Health Information Software, version 2 (DHIS2) for managing health information in resources-limited settings. It serves as a routine data source for collecting, validating, analysing, and presenting aggregate statistical data, capturing various facility-based health-related indicators relevant to integrated health information management activities. It is currently implemented in over 60 countries, allowing for their routine health data, particularly facility-based data, to be easily managed and visualised [17]. DHIS2 is especially prominent in LMICs; in 2009, it was incorporated into Bangladesh's Health Management Information Systems to collect data on the utilisation of health services [18] in order to provide data for regular facility monitoring and assessment. In relation to immunisation, the data collection process involves health care professionals recording immunisations during routine sessions, which are then entered into the facility's medical records and uploaded to the DHIS2 platform. As these records are maintained by trained professionals, they are considered reliable and less prone to (Appendix S1 in the **Online Supplementary Document**). It is important to note that DHIS2 provides data at the aggregate rather than the individual level, with monthly data collected from all available public facilities in Bangladesh’s eight divisions: Dhaka, Chattogram, Barishal, Khulna, Rajshahi, Rangpur, Mymensingh, and Sylhet. For our analysis, we extracted monthly division-level data from January 2017 to December 2021.

### Indicators

We examined the utilisation of childhood immunisation, considering three essential indicators: *Bacille Calmette-Guérin* (BCG) vaccination, the third dose of the pentavalent vaccine, and measles vaccination.

### Statistical analysis

We examined the yearly trend to show the annual changes in the number of vaccination administrations over time and compared the pre-pandemic period (2017–19) to the pandemic period (2020–21), whereby we computed the expected values for the years 2020 and 2021 by using the pre-pandemic period (2017–19) values as a basis. We then employed a linear regression model with the number of vaccination administrations as the outcome and the year as the regressor variable. We initially developed the model for the years 2017–19, after which we used it to project the expected values for both 2020 and 2021. We afterwards assessed the monthly trend on the pandemic period specifically to observe any fluctuations in the number of immunisations in order to pinpoint specific periods within the annual cycle that may have been more susceptible to disruption due to the COVID-19 pandemic. We used segmented regression, an approach commonly used in interrupted time series (ITS) studies, especially for analyses of how and if a given outcome has changed over time as a result of an intervention, policy, or programme. For example, a segmented Poisson regression is often selected to assess the disruptions in services caused by COVID-19, as the outcome of interest (‘number of services’ or ‘number of vaccination administrations’) is count data, making it well-suited for our analysis. It is also particularly effective for modelling rates that change over time, such as the dynamic impact of COVID-19 over time. The segmented approach also enables the analysis of interruptions and interventions, such as lockdowns or policy changes, which significantly affect service utilisation patterns. Understanding these effects is essential for our study, given the various disruptions caused by the pandemic. Through the utilisation of the segmented regression, we aimed to identify any shifts in the level of vaccination administration that were associated with particular time blocks. Several studies have used this approach to determine how an intervention affected an outcome of interest [10,19–21]. In our study, the variable ‘number of administration of vaccinations’, taken as count data, follows the Poisson distribution, justifying our use of segmented regression with Poisson distribution. We adjusted the segmented regression model for autocorrelation and stationarity to ensure the accuracy and reliability of our findings, and considered heteroscedasticity and autocorrelation consistent standard errors to account for autocorrelation when estimating standard errors [22]. We also used Fourier series, which are the linear com­binations of the sine and cosine functions [23] (Appendix S2 in the **Online Supplementary Document**). We reported the annual incidence rate ratio (IRR) with a 95% confidence interval (CI) in different regions in 2020 and 2021 compared to the reference period (2017–19), presenting the findings at both the national and division levels. We used Stata, version 15 (StataCorp LLC, College Station, TX, USA) to perform the analysis.

## RESULTS

**Figure 2:**
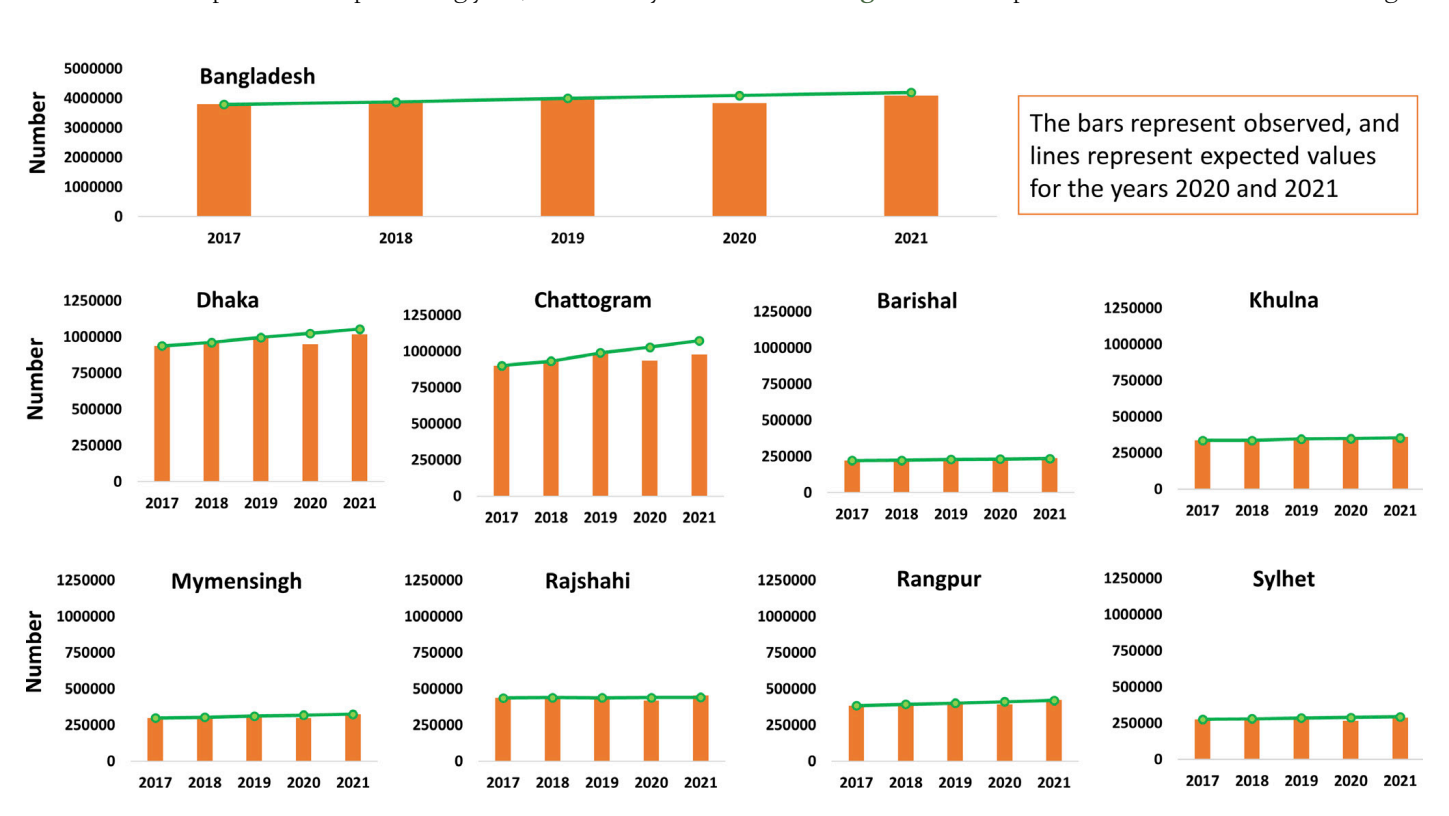
Trends in administration of BCG vaccination in childhood.

There was an upward trend in BCG vaccination in Bangladesh from 2017 to approximately four million vaccinations in 2019. A dip can then be observed to around 3.8 million in 2020, followed by a rebound in 2021, peaking at about 4.1 million BCG vaccinations (**Figure 2**). The actual observed values for BCG vaccine doses in 2020 and 2021, however, were lower than the expected values. This pattern remained consistent in Dhaka, Chattogram, and Sylhet. There was a consistent increase in the number of BCG vaccinations over the years in Dhaka, Barishal, Mymensingh, Rangpur, and Sylhet, with a temporary decrease observed in 2020. In 2021, these regions experienced a substantial rebound, showing a noticeable increase at similar or greater than the expected value.

**Figure 3:**
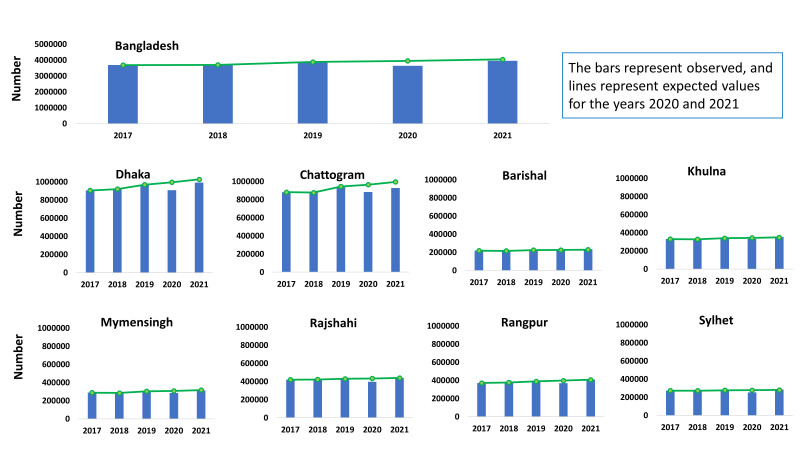
Trends in administration of third doses of the pentavalent vaccination in childhood.

The trend in the administration of the third dose of the pentavalent vaccine showed an overall growth, with a decline in 2020 compared to the preceding year, followed by a resurgence in 2021 (**Figure 3**). This pattern was consistent across all divisions. The 2020 values were lower than the expected values. In Barishal, Khulna, and Sylhet, the actual observed values exceeded the expected ones in 2021, while Chattogram had a lower number of administered third doses of pentavalent vaccine in 2021 compared to 2019.

**Figure 4:**
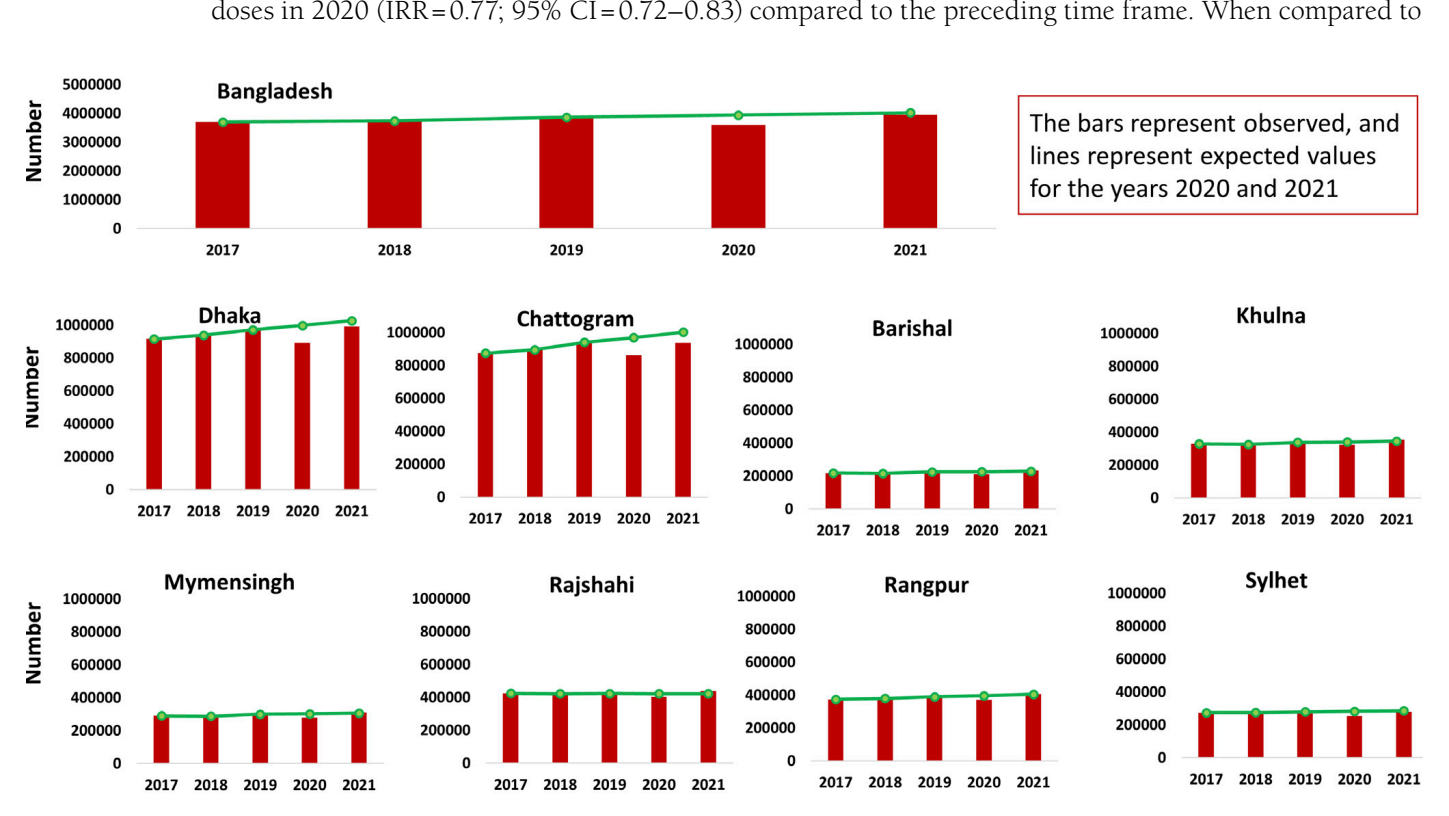
Trends in administration of measles vaccination in childhood.

The number of measles vaccinations increased over time from 2017 to 2019, with a decline in 2020 compared to the preceding year, followed by a rise in 2021 (**Figure 4**). This pattern remained the same throughout all of the divisions. The observed values were lower than the expected values in 2020. In 2021, meanwhile, the actual observed values in Dhaka, Chattogram, and Sylhet were lower than the expected value, while fewer doses of the measles vaccine were administered in Chattogram in 2021 even compared to 2019.

The number of administered BCG doses in Bangladesh was lower in 2020 (IRR = 0.83; 95% CI = 0.78–0.89) compared to the 2017–19 period and significantly higher in 2021 (IRR = 1.08; 95% CI = 1.07–1.08) (**Table 1**). Similar patterns were observed in every division except Chattogram, where the administration of BCG doses was significantly less frequent during 2020 but showed no significant change during 2021 compared to the 2017–19 period. Specifically, the division saw a notable decrease in the number of administered BCG doses in 2020 (IRR = 0.77; 95% CI = 0.72–0.83) compared to the preceding time frame. When compared to the previous period (2017–19), children from Mymensingh were less likely to receive BCG doses in 2020 (IRR = 0.92; 95% CI = 0.91–0.93) and more likely in 2021 (IRR = 1.22; 95% CI = 1.21–1.23).

**Table 1:** IRR of BCG vaccine administration during COVID-19 pandemic (2020 and 2021) compared with the pre-COVID-19 period (2017–19)

	2020		2021	
**Site**	**IRR (95% CI)**	***P*-value**	**IRR (95% CI)**	***P*-value**
Bangladesh	0.83 (0.78–0.89)	<0.001	1.08 (1.07–1.08)	<0.001
Dhaka	0.82 (0.75–0.89)	<0.001	1.02 (1.02–1.03)	<0.001
Chattogram	0.77 (0.72–0.83)	<0.001	1.00 (0.93–1.07)	0.946
Barishal	0.90 (0.89–0.91)	<0.001	1.16 (1.15–1.17)	<0.001
Khulna	0.87 (0.86–0.88)	<0.001	1.10 (1.09–1.11)	<0.001
Mymensingh	0.92 (0.91–0.93)	<0.001	1.22 (1.21–1.23)	<0.001
Rajshahi	0.82 (0.77–0.88)	<0.001	1.12 (1.04–1.20)	0.001
Rangpur	0.90 (0.90–0.91)	<0.001	1.16 (1.09–1.24)	<0.001
Sylhet	0.81 (0.73–0.89)	<0.001	1.09 (1.04–1.14)	<0.001

CI – confidence interval, IRR – incidence rate ratio

There was an observable variation in the number of administered pentavalent third doses across different sites in Bangladesh (**Table 2**). The administration of third doses of pentavalent in Bangladesh was lower in 2020 (IRR = 0.82; 95% CI = 0.76–0.89) and higher in 2021 (IRR = 1.14; 95% CI = 1.10–1.17) compared to the reference period (2017–19). In Sylhet, the number of administered third doses of pentavalent vaccines was too low in 2020 (IRR = 0.75; 95% CI = 0.67–0.84). However, the number of administered pentavalent third doses in 2021 increased significantly in all divisions. For example, in Rangpur, it was higher in 2021 (IRR = 1.21; 95% CI = 1.16–1.25) compared to the 2017–19 period.

**Table 2:** IRR of pentavalent third dose administration during COVID-19 pandemic (2020 and 2021) compared with the pre-COVID-19 period (2017–19)

	2020		2021	
**Site**	**IRR (95% CI)**	***P*-value**	**IRR (95% CI)**	***P*-value**
Bangladesh	0.82 (0.76–0.89)	<0.001	1.14 (1.10–1.17)	<0.001
Dhaka	0.77 (0.69–0.85)	<0.001	1.10 (1.07–1.14)	<0.001
Chattogram	0.77 (0.70–0.86)	<0.001	1.09 (1.05–1.13)	<0.001
Barishal	0.95 (0.89–1.01)	0.080	1.12 (1.07–1.16)	<0.001
Khulna	0.92 (0.87–0.98)	0.005	1.07 (1.02–1.12)	0.009
Mymensingh	0.81 (0.74–0.89)	<0.001	1.19 (1.14–1.24)	<0.001
Rajshahi	0.86 (0.80–0.93)	<0.001	1.19 (1.15–1.23)	<0.001
Rangpur	0.95 (0.90–1.00)	0.036	1.21 (1.16–1.25)	<0.001
Sylhet	0.75 (0.67–0.84)	<0.001	1.02 (1.01–1.04)	0.010

CI – confidence interval, IRR – incidence rate ratio

In 2020 and 2021, the number of administered doses of measles vaccines decreased across all divisions compared to the 2017–19 period (**Table 3**). Notably, the IRR was higher in 2021 than in 2020 in all divisions except Khulna, indicating an increase in the number of administrations. The administration of measles vaccine doses in Bangladesh was found to be significantly lower in both 2020 (IRR = 0.85; 95% CI = 0.78–0.93) and 2021 (IRR = 0.94; 95% CI = 0.94–0.94) when compared to the 2017–19 period. Across all divisions, the administration of measles vaccines was the lowest in Sylhet in 2020 (IRR = 0.79; 95% CI = 0.71–0.88) and in 2021 (IRR = 0.84; 95% CI = 0.84–0.85) compared to the 2017–19 period.

**Table 3:** IRR of measles vaccine administration during COVID-19 pandemic (2020 and 2021) compared with the pre-COVID-19 period (2017–19)

	2020		2021	
**Site**	**IRR (95% CI)**	***P*-value**	**IRR (95% CI)**	***P*-value**
Bangladesh	0.85 (0.78–0.93)	<0.001	0.94 (0.94–0.94)	<0.001
Dhaka	0.80 (0.73–0.89)	<0.001	0.94 (0.94–0.95)	<0.001
Chattogram	0.83 (0.76–0.91)	<0.001	0.94 (0.87–1.02)	0.146
Barishal	0.87 (0.78–0.96)	0.007	0.99 (0.98–1.00)	0.044
Khulna	0.96 (0.95–0.97)	<0.001	0.94 (0.93–0.95)	<0.001
Mymensingh	0.80 (0.71–0.90)	<0.001	0.94 (0.93–0.95)	<0.001
Rajshahi	0.95 (0.89–1.02)	0.166	0.97 (0.96–0.98)	<0.001
Rangpur	0.91 (0.85–0.99)	0.021	0.96 (0.96–0.97)	<0.001
Sylhet	0.79 (0.71–0.88)	<0.001	0.84 (0.84–0.85)	<0.001

CI – confidence interval, IRR – incidence rate ratio

**Figure 5:**
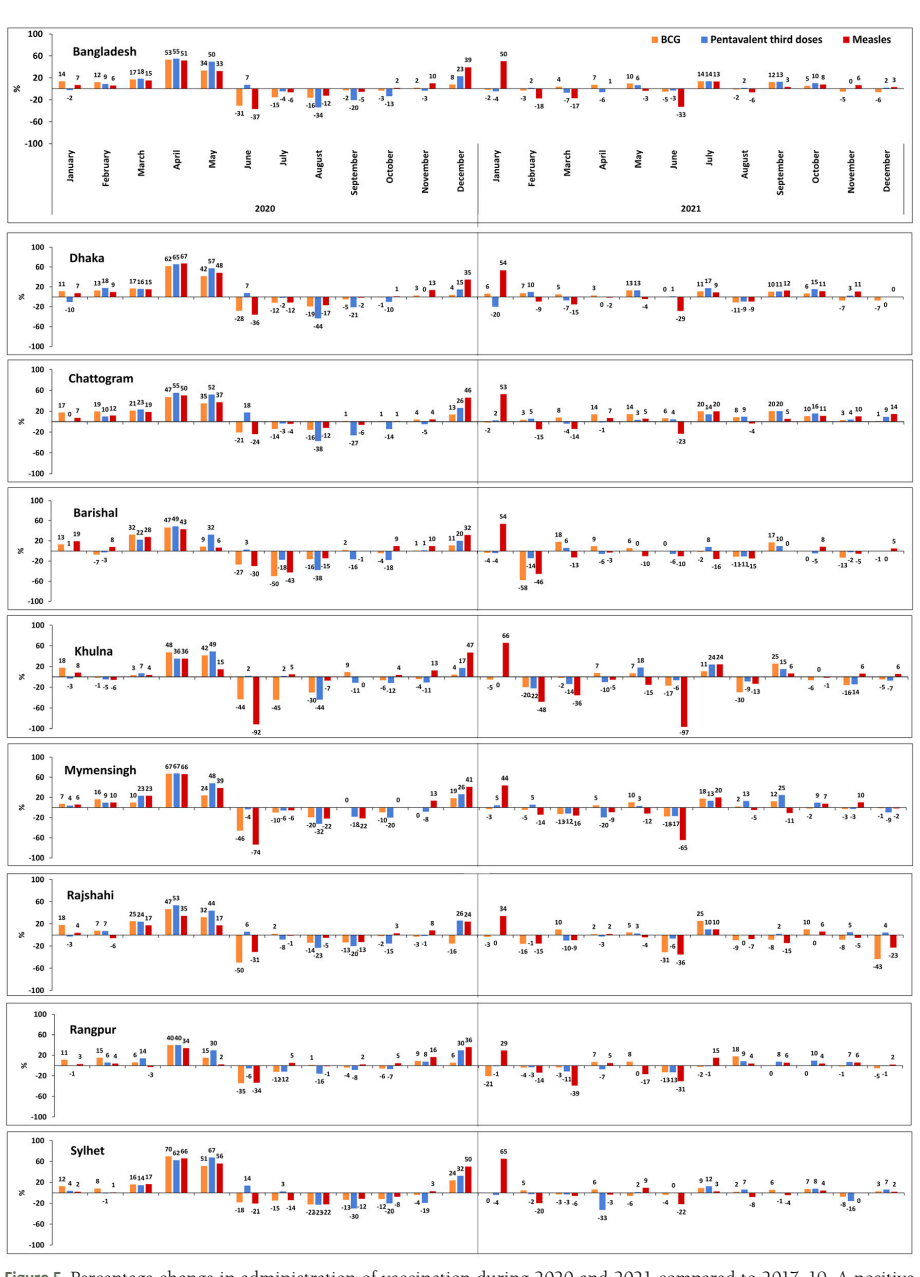
Percentage change in administration of vaccination during 2020 and 2021 compared to 2017–19. A positive value indicates a decline in vaccine administration during 2020 and 2021 relative to the preceding period of 2017–19, while a negative value indicates an increase.

At the national level, the most substantial decreases occurred in April (BCG: 53%; pentavalent third doses: 55%; and measles: 51%) and May (BCG: 34%; pentavalent third doses: 50%; and measles: 33%) of 2020 compared to the 2017–19 period (**Figure 5**). While there was a further increase in the administration of these vaccines starting in June 2020, levels began to decline again at the end of the year, but recovered by February 2021. It is also evident that the average values of the administered of BCG, pentavalent third doses, and measles vaccines during the 2017–19 period remained relatively stable, with slight fluctuations observed across different months. Conversely, there was a substantial drop in the administration of vaccinations for several months of 2020, with fewer fluctuations observed in 2021 (Figures S1–3 in the **Online Supplementary Document**). A similar pattern was found for all vaccine types across all divisions, with a noticeable decline observed in April 2020 (**Figure 5**), whereby the largest reduction in BCG vaccination administration (70% decrease) occurred in Sylhet. The largest reduction in the number of administered pentavalent third doses was observed in Mymensingh (67%), while this drop was slightly lower (62%) in Sylhet. However, in April 2020, the distribution of the measles vaccine dropped by 67% in Dhaka and 66% in Sylhet compared to the 2017–19 period. Sylhet once more experienced a higher decline of 65% in the number of administered measles vaccines in January 2021.

Across the different regions, divisions such as Khulna shows some recurring patterns on an annual basis both pre-pandemic and in 2021 (Figure S1 in the **Online Supplementary Document**). Before the pandemic, the immunisation administration in most regions appeared to be stable, with slight fluctuations. During the pandemic, particularly from April 2020 onward, there was a noticeable drop across almost all regions and vaccine types. For example, the usual peaks for measles vaccinations were either delayed or less pronounced in January 2021, indicating a deviation from the expected seasonal patterns. (Figure S3 in the **Online Supplementary Document**)

## DISCUSSION

To our knowledge, this is the first study to explore the COVID-19 pandemic’s impact on child vaccination across sub-national areas in Bangladesh. Prior analyses outlined a decline in health service utilisation at the national level in Bangladesh [9]. Here we found a reduction in the average number of administered vaccines at the national level, especially during the months of April and May 2020. We also found that the pandemic's effects varied across different geographical regions, highlighting the regional dynamics influencing health care access during a global health crisis. Regarding vaccination administration during the COVID-19 pandemic, Sylhet and Chattogram emerged as the two most severely impacted regions in Bangladesh. Dhaka, the capital city of Bangladesh, also saw a significant reduction in measles vaccine administration in the initial months of the pandemic. This disparity raises important questions about the regional dynamics influencing health care delivery and access, especially in the event of a worldwide pandemic.

The findings show that Sylhet experienced the largest reduction (70% decrease) in BCG vaccine administration in April 2020 compared to the pre-COVID-19 period. Sylhet, once more, witnessed a higher decline, with a 65% reduction in measles vaccine administration in January 2021. Multiple factors could explain these differential regional effects. A study conducted using 2014 Bangladesh Demographic and Health Survey (BDHS) reported that Sylhet had considerably fewer cases of full vaccination (defined as the receipt of one dose of BCG, three doses of pentavalent vaccine, three doses of oral polio vaccine (OPV), and one dose of measles) than all other regions [24]. It also had the lowest vaccine coverage for all vaccine series. This striking disparity, observed prior to the COVID-19 pandemic, hints at a complex web of factors affecting health care access and public awareness. A reason for this disparity may be the lack of public awareness and education about the importance of vaccinations. Moreover, regions with weaker health system might experience lower vaccination rates, especially during a public health crisis like the COVID-19 pandemic. Of all divisions, Sylhet is the third in terms of having slum-dwellers (n = 127 584), while Dhaka is the first [15]. Despite this, Sylhet faces disparities in health care access and geographic constraints. The impact of the pandemic on its high slum population, coupled with lower awareness among slum-dwellers, contributes to a lower number of vaccination administrations in the region. Sylhet is also a geographically distinct region that is relatively far from Dhaka, and its topography may have posed challenges for timely vaccine distribution and administration during COVID-19, where the existence of difficult-to-reach remote areas and transportation constraints might have resulted in vaccine delivery delays. These findings are consistent with previous studies that found negative impacts on overall [14,25,26] and disease-specific service utilisation [14]. As a result of the global pandemic's burden on health care systems and the subsequent reallocation of personnel and resources to address COVID-19, essential health services have become less accessible [13]. These pre-existing and pandemic-related factors combined to create a perfect storm of difficulties for Sylhet's health care system. These geographical obstacles highlight the necessity for innovative approaches to ensure the timely delivery of vaccinations, particularly in times of public health emergency.

All divisions of Bangladesh reported lower vaccination numbers in 2020 compared to 2017–19. However, while most divisions rebounded in 2021, with numbers exceeding 2019 levels, Chittagong remained an exception, maintaining a decline in vaccine administration even below the 2019 figure. This can be attributed to several interrelated factors. An alarming increase in the number of deaths in the post-lockdown period (August 2020 to January 2021) instilled a deep sense of fear among the division’s population [27]. This fear of contracting the virus and the potential dire consequences may have discouraged families from timely vaccinating their children. Additionally, existing data suggested that the COVID-19 infection rate in people from urban areas such as Chittagong was higher than in rural villages, a trend consistent with many urban settings worldwide [28]. Moreover, the high population density and congested infrastructures in cities like Dhaka and Chittagong created an environment conducive to virus transmission, raising concerns among residents [27]. In the context of Chittagong, the arrival of over 723 000 Rohingya refugees in Cox’s Bazar, a remote region of the city, placed additional strain on the health care infrastructure since 25 August 2017 [29]. The Rohingya refugees settled in crowded camps with limited access to essential services, including health care, education, food, clean water, and proper sanitation [30]. Low levels of education and public awareness among these refugee populations, coupled with the challenging living conditions, likely contributed to lower vaccination rates. Differences in health care infrastructure and resources across regions can exert a notable influence on vaccination programmes. This challenging environment likely contributed to the lower administration of vaccinations in recent times in Chittagong, particularly. Furthermore, Chittagong’s population includes a higher percentage of economically disadvantaged individuals and families, who may have faced greater challenges in accessing health care services, including vaccinations. A study conducted in 2020 reported an 80% drop in the per capita daily income of urban slum and rural poor populations due to the countrywide lockdown imposed by the government to curb the spread of COVID-19 [31].

Dhaka, meanwhile, experienced the highest drop in the administration of measles vaccines at the beginning of the COVID-19 pandemic. These observed disparities can be attributed to several factors. Variations in the spread of COVID-19 and the preventive measures implemented across regions could explain the regional heterogeneity in service disruptions. A study conducted in 2020 reported that Dhaka, with a population of nearly 20 million people, was the country’s epicentre of COVID-19 infections [27] which can be a critical driver for the significant impact on health care services. Thus, the implementation of COVID-19 preventive measures, including movement restrictions and social distancing, was primarily concentrated in urban regions [28]. However, implementing physical distancing practices in densely populated urban slum areas posed significant challenges [32]. Dhaka is also home to over one million slum dwellers and marginalised communities living in close proximity, putting them at risk due to inadequate facilities for maintaining personal hygiene [33,34]. The lack of awareness, combined with the fear of infection, likely led to reduced vaccine uptake in these areas. Furthermore, the reallocation of health care workers to pandemic response efforts may have had an impact on routine vaccination services in these areas. This shift in health care workforce resources may have contributed to the observed disparities in vaccine utilisation in Dhaka during the pandemic's early stages.

Compared to 2017–19, the most significant drop in vaccine administration in Bangladesh occurred in April and May 2020. For the first several months, the COVID-19 pandemic had negative effects on immunisation services, which is consistent with several other studies [11,35]. The drop in vaccination rates observed during the early pandemic phase in 2020 compared to the preceding years of 2017–19 can be attributed to the convergence of several factors. Following the initial declaration of the COVID-19 pandemic, extensive lockdown measures and transit restrictions were implemented, adversely affecting the logistics and supply chain of essential pharmaceuticals, including vaccines. These restrictions posed a serious barrier to the delivery of health care services, which included regular vaccinations [36]. Similarly, the use of widespread quarantine measures has unintentionally led to children not receiving their regular immunisations. Simultaneously, a lack of appropriate personal protection equipment for health care professionals, inadequate staffing levels in health care facilities, and other operational factors impacted on the delivery of health care services [36]. Several countries, including Bangladesh, had trouble setting up their health care systems to handle the pandemic in an efficient manner, hence intensifying disruptions in vaccination [36]. According to a study conducted in 11 sub-Saharan African countries, the COVID-19 pandemic limited service utilisation for 18% of study participants, due to travel restrictions, health facility closures, and fear of contracting the virus [37]. During the COVID-19 outbreak in Rwanda, the use of maternal and child health services across all four categories – antenatal care, deliveries, postnatal care, and vaccinations – dropped significantly, with analyses suggesting that these decreases might be exacerbated by pre-existing challenges, including poor service quality, poor road conditions, disrupted supply deliveries to health facilities, and insufficient infrastructure for the domestic production of medical supplies [35]. Even the WHO release of guidelines advising a temporary stoppage of mass vaccination campaigns has been a major contributor to the drop-in vaccination rates [38].

We observed an improvement in vaccination rates, particularly in the latter half of 2020, following the initial impact of the pandemic. This trend is in line with findings from other studies [36,39]. Bangladesh’s nationwide lockdown was extended multiple times until 30 May 2020; as lockdown restrictions started to ease in June 2020, vaccination rates seemingly recovered [36]. According to a global modelling study, the second half of 2020 showed signs of recovery in childhood immunisation in many countries, with global immunisation rates beginning to approach expected estimates by December 2020 [40]. For example, a prior study reported that in Southeast Asia, East Asia, and Oceania, the estimates for both the third dose of diphtheria-tetanus-pertussis and the first dose of measles-containing vaccine indicated that monthly doses were delivered at or above expected levels during the latter half of 2020 [40].

However, here we found that levels of administration of vaccination began to decline again at the end of 2020, with a significant drop observed in the number of administered measles vaccines, particularly in January 2021. Notably, health workers’ went on a strike on 26 November 2020, inclusive of those responsible for administering and implementing national immunisation programmes. The protests by health workers demanding a pay increase halted routine immunisation across the country, impacting millions of lives. As a result, the measles vaccination campaign initially scheduled for 6 December was postponed to 12 December. Therefore, this drop-in measles vaccination is especially notable because the strike occurred immediately before a national measles vaccination campaign [41]. It is, however, worth noting that the measles vaccinations delivered within this nationwide campaign in January 2021 were reported to a separate reporting system that was not integrated with the regular Expanded Program on Immunisation information system [9].

There could have been a number of reasons for the decline in vaccination rates in 2020, such as supply chain disruptions, lockdowns, movement restrictions, and worries about infection [42]. Alternatively, various factors such as maintaining trust, implementing equity-focused strategies, creating innovative methods of administering vaccines to ensure the availability of vaccines in both rural and urban communities, establishing climate resilient systems, enhancing readiness for outbreaks, optimising vaccine supplies, and additional strategies outlined in The Immunisation Agenda 2030 have significantly contributed to the resurgence of vaccine utilisation from 2021 onwards [43].

The Government of Bangladesh implemented various strategies to address the adverse impacts of the COVID-19 pandemic on essential health services. The Bangladesh Preparedness and Response Plan for COVID-19, formulated in March 2020, emphasised the importance of maintaining essential health and nutrition services during the pandemic [44]. Bangladesh imposed lockdowns three times: from 26 March to 30 May 2020; from 28 June to 14 July 2021; and from 23 July to 5 August 2021 [45]. However, studies have shown that the government’s lockdown measures were not effectively enforced in rural areas, and movement restrictions gradually eased without clear guidelines or a sound scientific basis [46]. Additionally, there was a lack of coordination among different government agencies when it came to emergency health care and crisis management on the ground, which severely compromised the availability of essential health services during the pandemic [46].

### Implications for policy and practice

The disparities that have been noted here underscore the necessity for focussed, region-specific interventions aimed at guaranteeing equitable access to health care services, specifically in areas where vulnerabilities exist. Several factors, including economic disparities, public awareness, geographic obstacles, and pandemic-related concerns, affect vaccination coverage rates. A diverse set of approaches is needed to address these challenges, such as increasing public awareness campaigns, ensuring timely vaccine distribution, and improving health care infrastructure, but also focussing on region-specific decision-making. For example, timely resource allocation is key in the case of Sylhet, where it could help improve the division’s overall health system infrastructure, including the expansion and enhancement of health care facilities and clinics, especially in the context of the allocation of government and donor resources in remote and underserved areas of Sylhet. Developing community-based programmes that engage local leaders and influencers can help raise awareness about the importance of vaccination. In Chittagong, where vaccination rates remained low even in 2021, efforts should be directed towards improving health care infrastructure and accessibility. There is also an urgent need to improve the preparedness of the health care workforce in order to adequately address and manage outbreaks and crises. Targeted public awareness campaigns are needed to address the specific concerns and misconceptions about vaccines in the urban and peri-urban populations, especially in densely populated and low-income areas. To ensure health care services are maintained even in densely populated cities like Dhaka, health care practitioners must be provided with appropriate personal protective equipment and adequate staff numbers. Investing in making health care infrastructure more resilient to future crises is crucial, including retrofitting hospitals and clinics to handle increased patient loads and ensuring that health care delivery is not disrupted by city-wide lockdowns or other emergency measures. To improve crisis management and emergency medical care, there needs to be better coordination between various government agencies and stakeholders. This will support the continuation of essential health services in times of crisis.

### Strengths and limitations

Our study included a rigorous analysis of immunisation trends in Bangladesh, including a large sample size from DHIS2, which provides detailed regional data and is known for its robustness and completeness in terms of vaccination data [47], allowing us to effectively draw conclusions at the regional level. Our exploration of annual and monthly trends provides a clear understanding of the fluctuations in immunisation throughout the pandemic and helps to point out seasonal patterns and specific time periods that are critical for public health planning and response. In addition, we employed a rigorous, but simple method – a segmented regression model with Poisson distribution which accounts for count data characteristics and addresses autocorrelation and stationarity, enhancing the robustness of our analysis.

However, we used routinely collected data from DHIS2, which is provided at an aggregate and not at an individual level; this means we did not have access to detailed demographic or socioeconomic information about the children or their parents or individual attitudes towards immunisation. Future studies with access to individual-level data could provide deeper insights into the demographic and socioeconomic factors influencing immunisation rates and capture individual attitudes towards immunisation. However, while valuable, DHIS2 may have limitations related to data quality, completeness, and accuracy; for example, the availability of the ‘SKIP’ for any indicator [18] allows users to skip certain questions or indicators in a data entry form, resulting in data incompleteness. A problem may also arise with the timeliness of the data if it is not entered into the system routinely. These factors may have led to underestimation or overestimation of health indicators. Although data accuracy and completeness remain an issue, all users are encouraged to use DHIS2; for example, Bangladesh’s HMIS division encourages the best-performing region through annual rewards to increase DHIS2 usage [18]. Lastly, we used data extracted from public facilities exclusively, which could lead to potential gaps in our understanding of the utilisation of health care services. The vast majority of private facilities in the country do not report to the national HMIS [18], leading to incompleteness of health information, which can impede effective health planning and policy-making. It is essential to acknowledge that private health care facilities can play a significant role in vaccination services. However, the observed disruption in 2020 is not likely to be attributable solely due to a shift to private services, as public facilities remained operational throughout the pandemic.

## CONCLUSIONS

We observed substantial variations in vaccination administration across different regions, highlighting the complexities of health care access during a global health crisis. After an initial disruption caused by the pandemic, our data show that immunisation rates have been increasing, especially in the second half of 2020. COVID-19 had a severe and universal effect on all services in Bangladesh, with the health worker strikes later in the year further exacerbating the challenges for vaccination services. There has, however, been a notable increase in the administration of vaccinations in 2021. Nevertheless, regional disparities in vaccination coverage were evident, with Sylhet and Chattogram experiencing the most noticeable effects. Dhaka, Bangladesh’s capital city, saw the greatest drop in the number of administered measles vaccines at the start of the COVID-19 pandemic. Policymakers should adapt strategies to address the unique needs of each region.

## Additional material


Online Supplementary Document
