# Supplementary material for: Unveiling the dimension of regional disparities: Assessing the disruption of immunisation services by COVID-19 in Bangladesh
Source: J Glob Health. 2024 Oct 25;14:05028. doi: 10.7189/jogh.14.05028 (PMC11505652; doi:10.7189/jogh.14.05028)
Supplement: Online Supplementary Document [file jogh-14-05028-s001.pdf]

Supplementary materials:

Figure S1: Annual and monthly trends in administration of BCG vaccination in childhood

Bangladesh

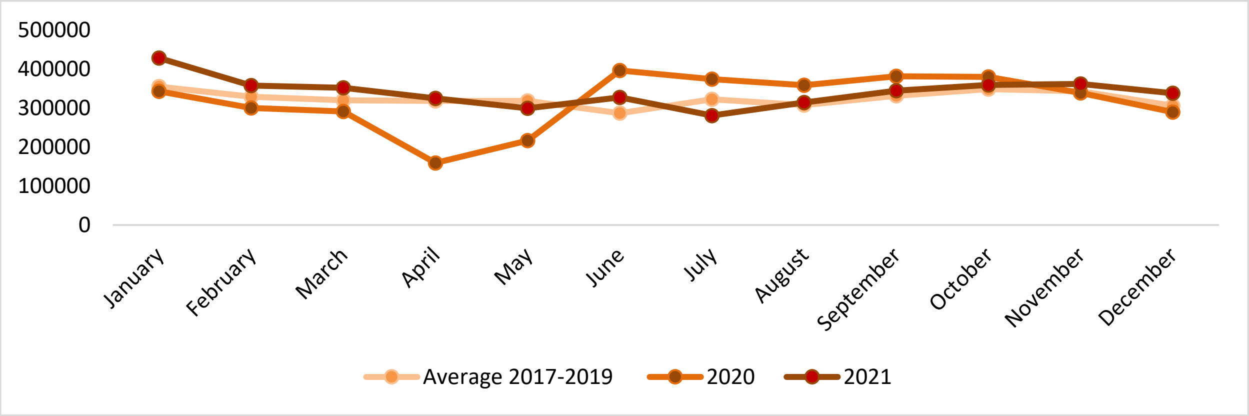

Dhaka

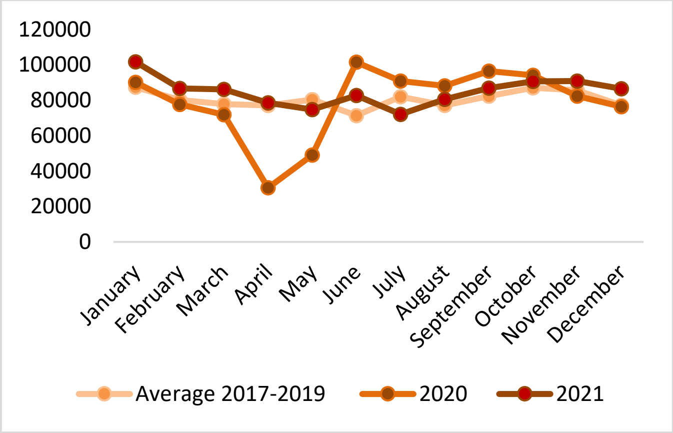

Chattogram

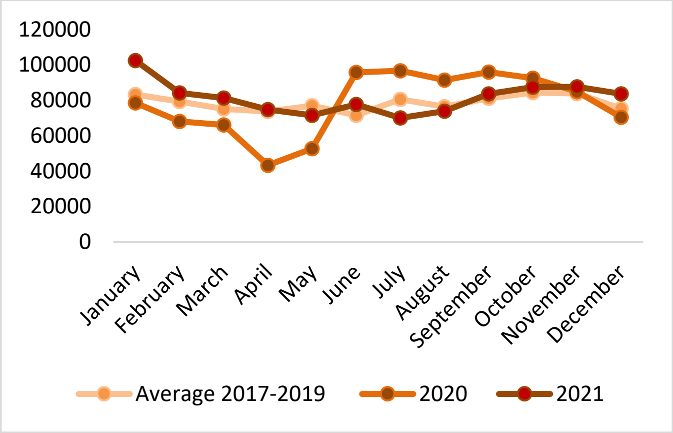

Barisal

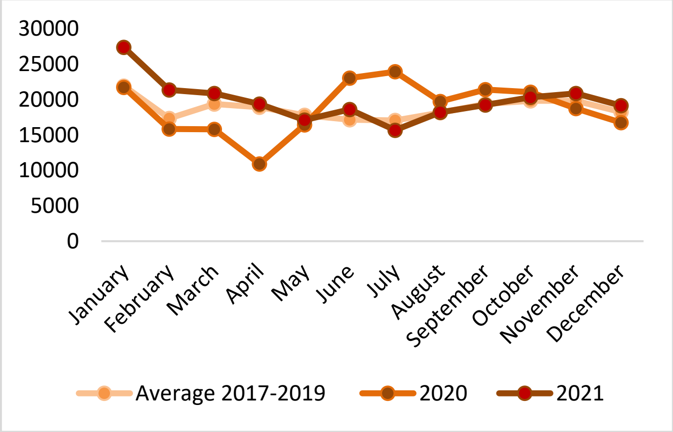

Khulna

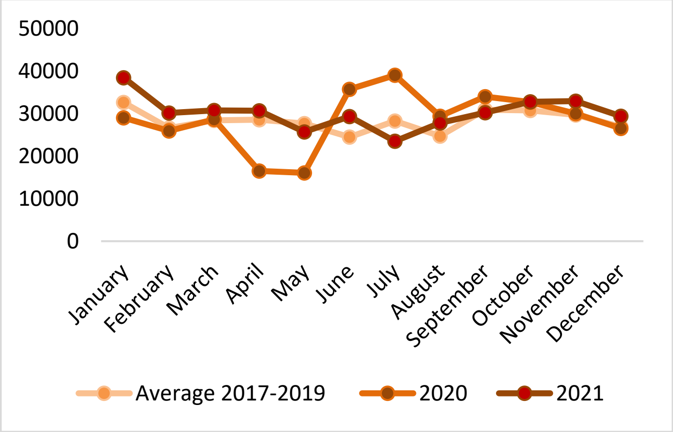

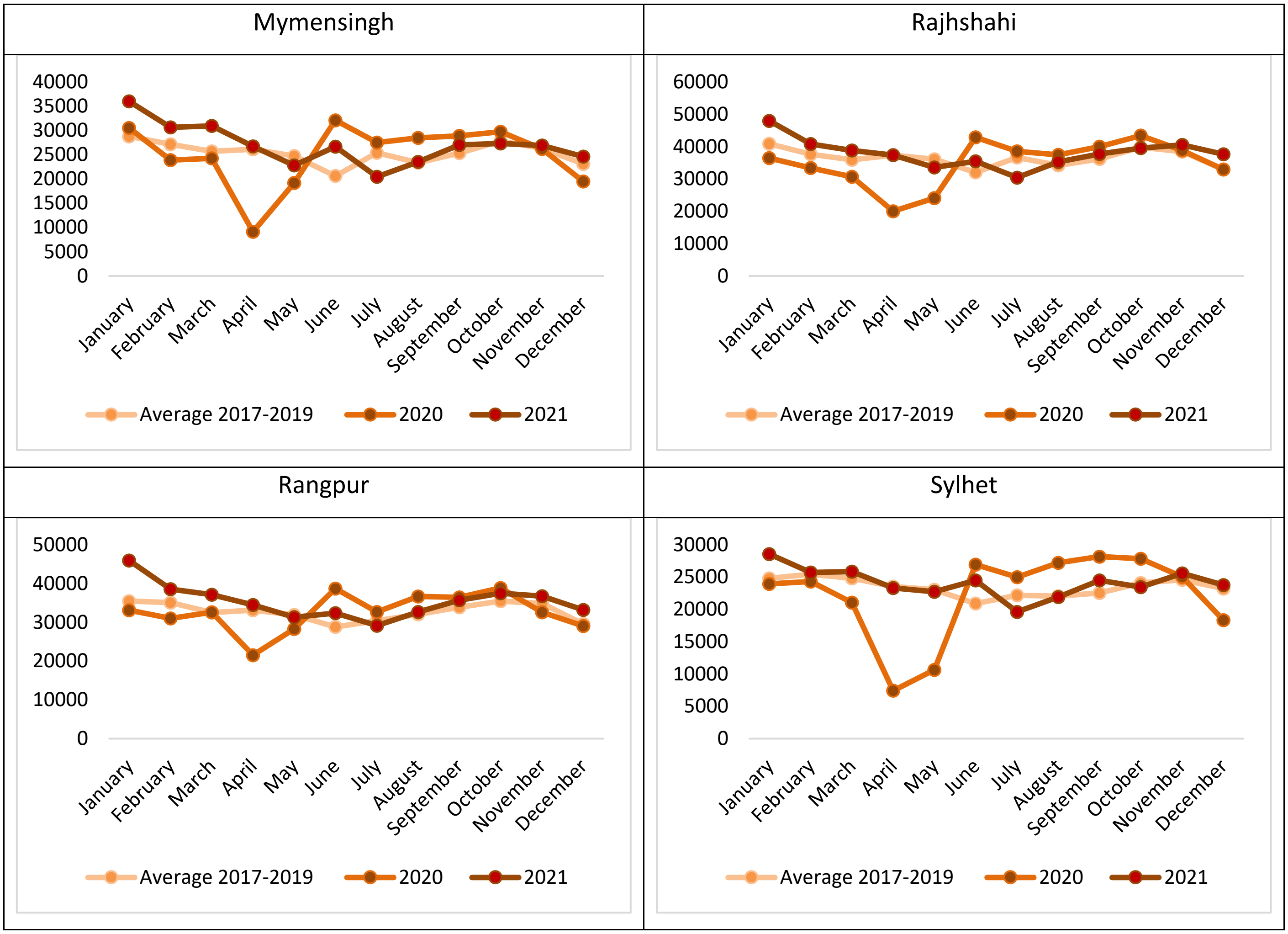

Figure S2: Annual and monthly trends in administration of Pentavalent third doses vaccination in childhood

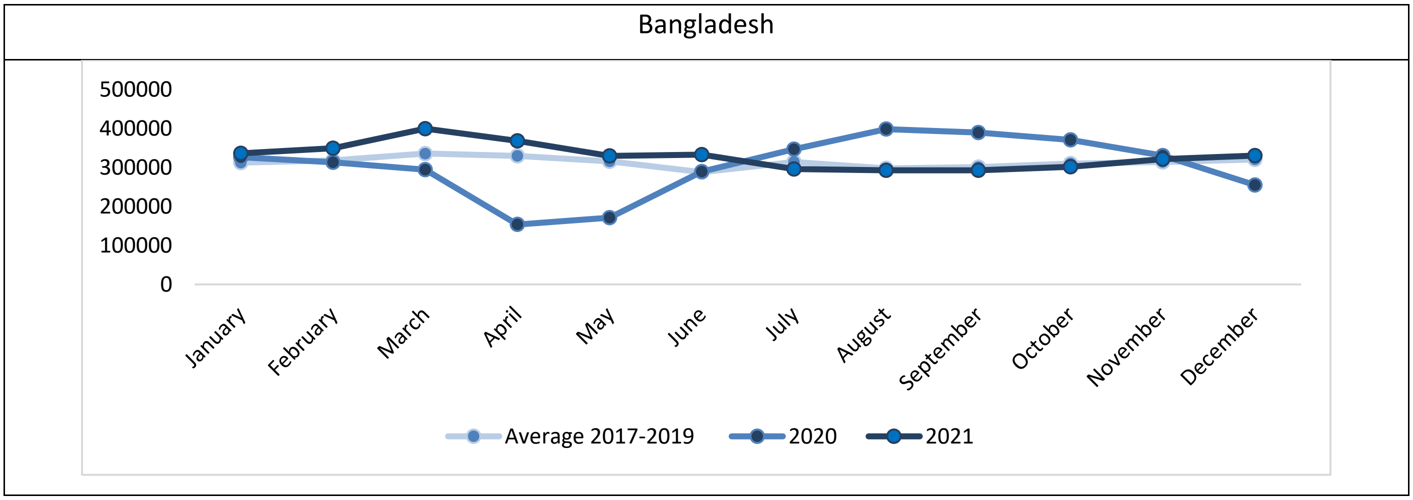

Dhaka

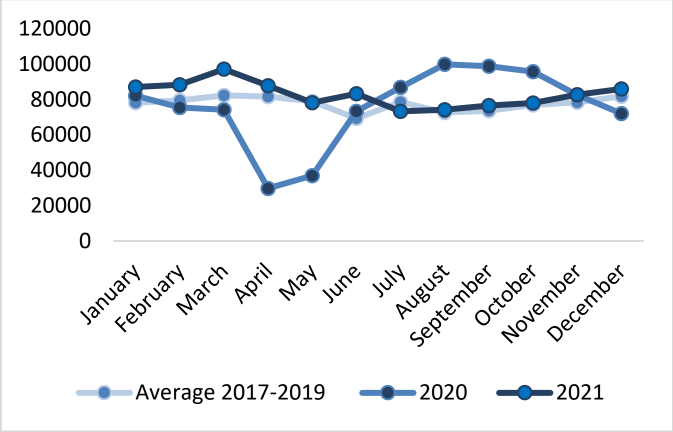

Chattogram

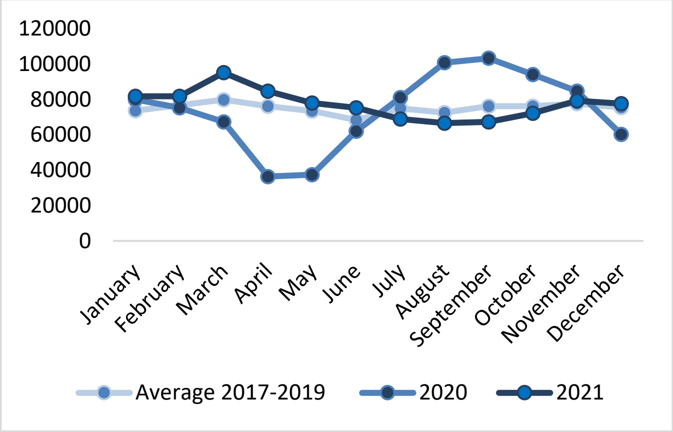

Barisal

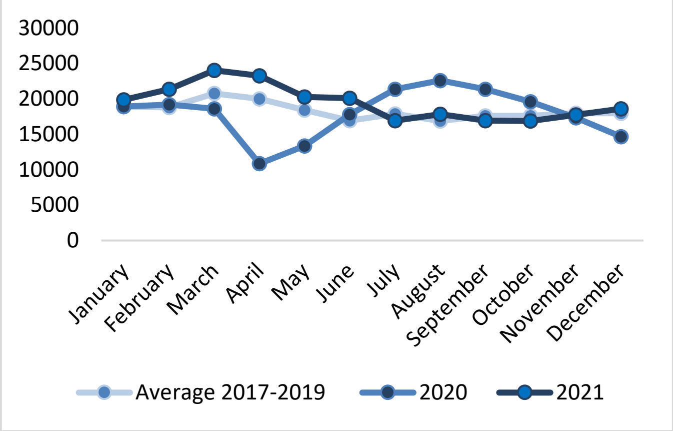

Khulna

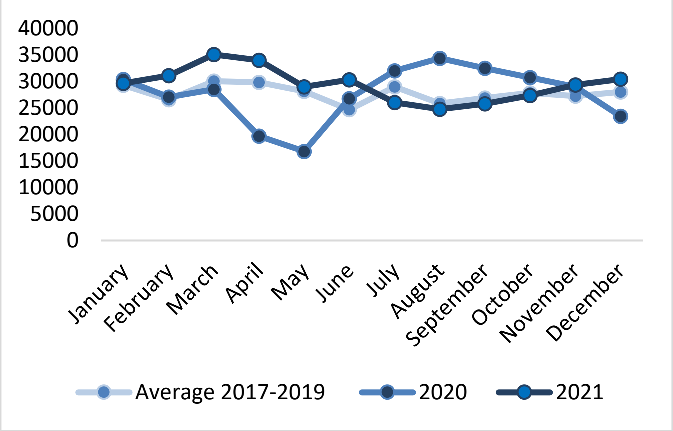

Mymensingh

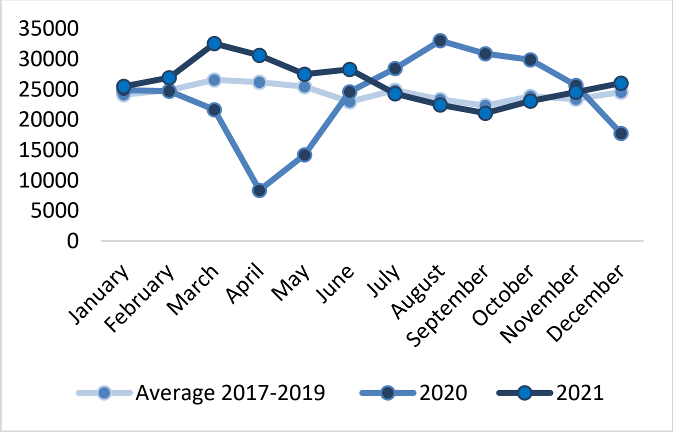

Rajshahi

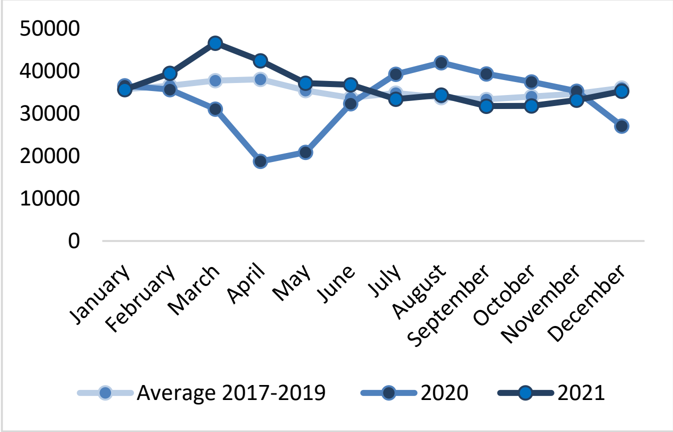

Rangpur

Sylhet

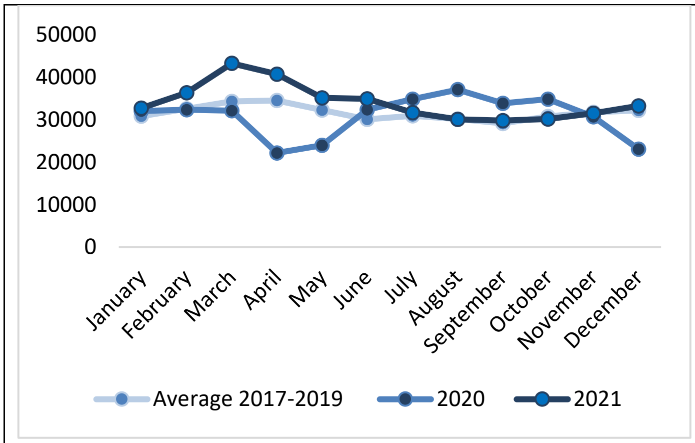

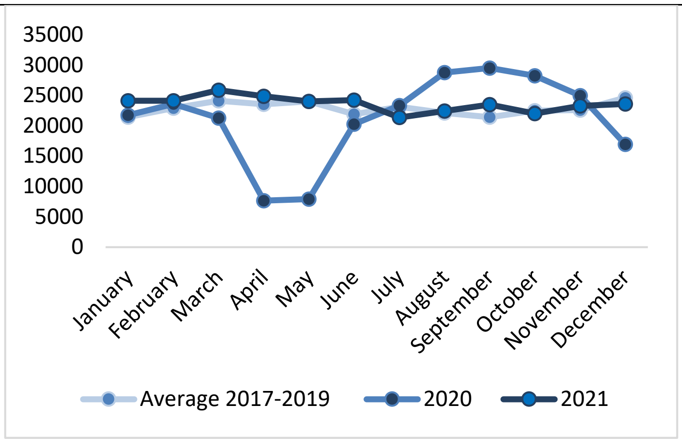

Figure S3: Annual and monthly trends in administration of Measles vaccination in childhood

Bangladesh

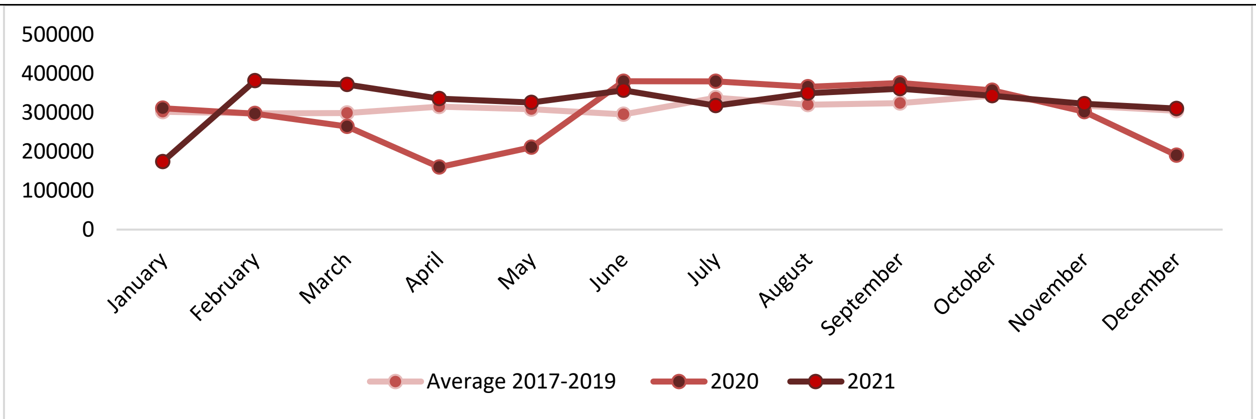

Dhaka

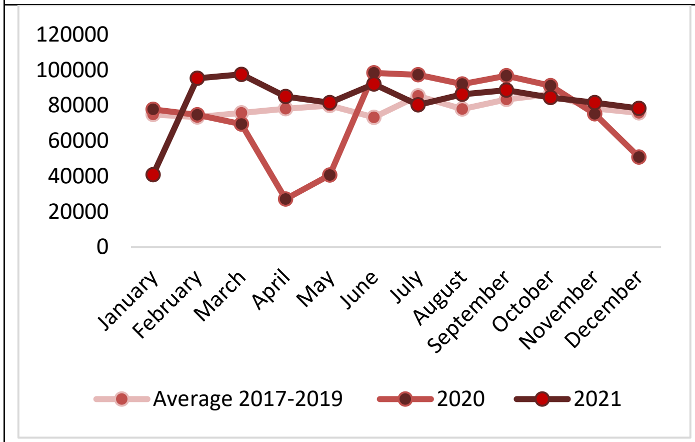

Chattogram

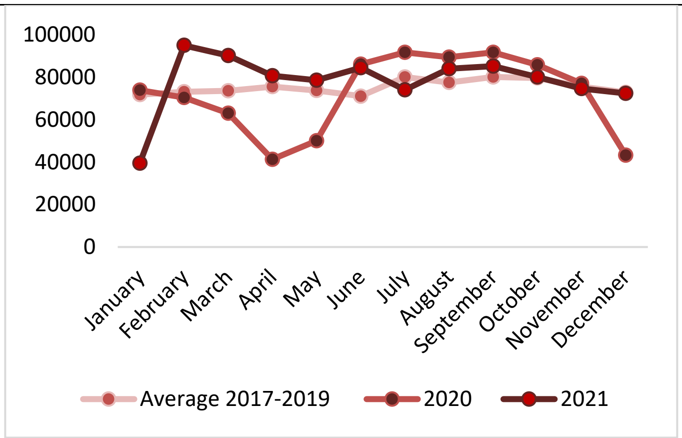

Barisal

Khulna

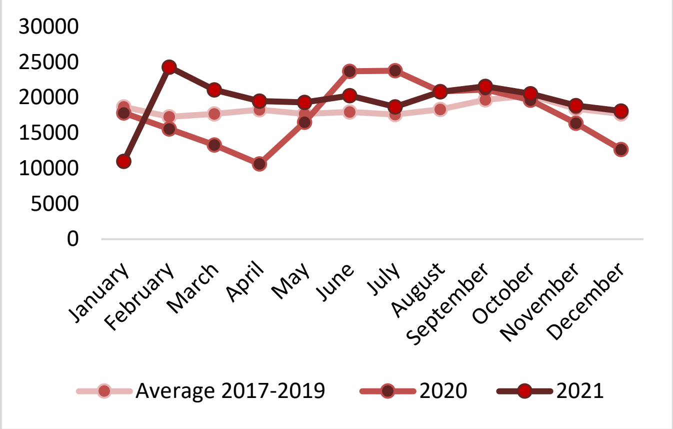

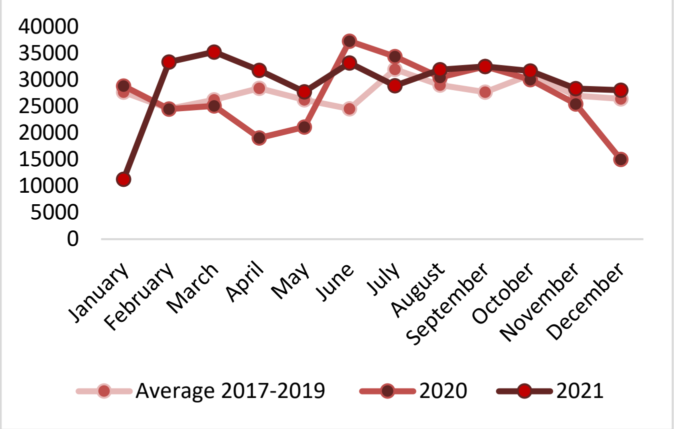

Mymensingh

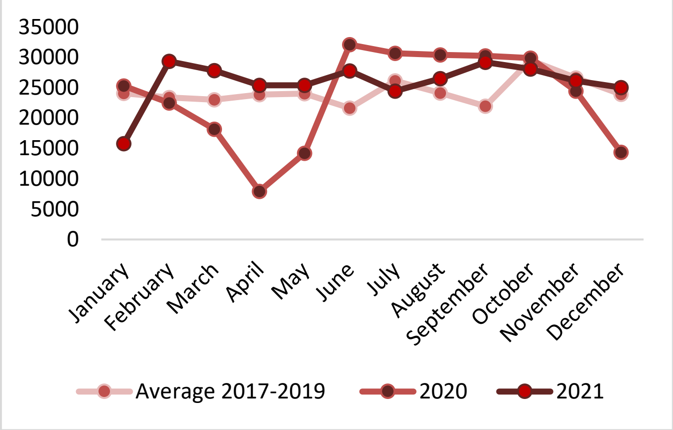

Rajshahi

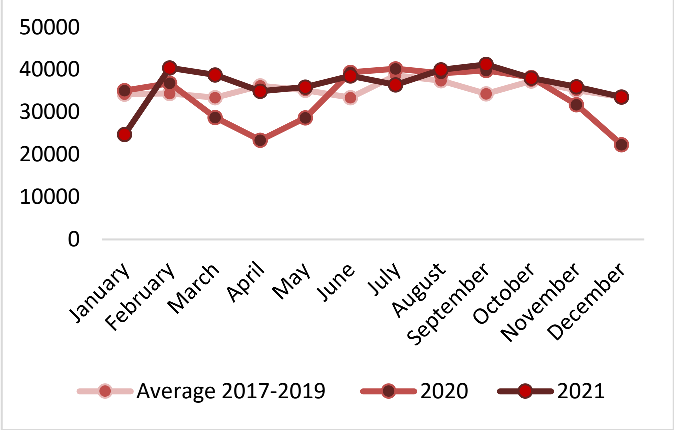

Rangpur

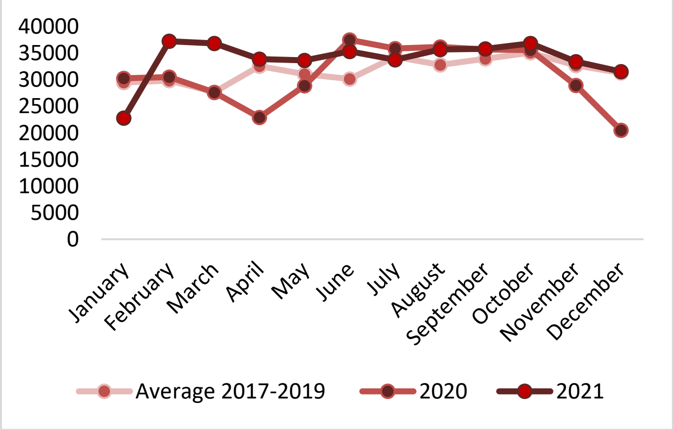

Sylhet

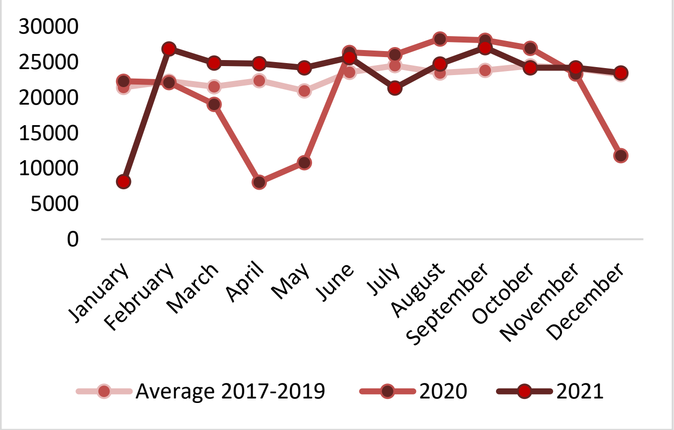

Appendix S1: Data quality of DHIS2

Data quality can be assessed by completeness and timeliness of facility reporting. DHIS2 provides information on the completeness and timeliness of collected reports. Children immunisation related indicators are included in the “EPI Infant and Women Dataset” report.

Completeness: The completeness of a particular report or dataset, such as the reported "EPI Infant and Women Dataset", within the DHIS2 database was assessed by comparing the total number of reported health facilities/community workers in the DHIS2 database to the total number expected to report. As of December 2021, the completeness of "EPI Infant and Women Dataset" in DHIS2 was nearly 100%, suggesting that the quality of DHIS2 data for immunisation indicators is almost complete.

Timeliness: The timeliness of reporting in DHIS2 was evaluated based on the availability of reports within a fixed reporting date. During our study period, the timeliness of "EPI Infant and Women Dataset" reports was below 90%.

Figure S4: Snapshot of data quality status of "EPI Infant and Women Dataset" report from DHIS2 webpage

"EPI Infant and Women Data set (Form-1&2)" completeness

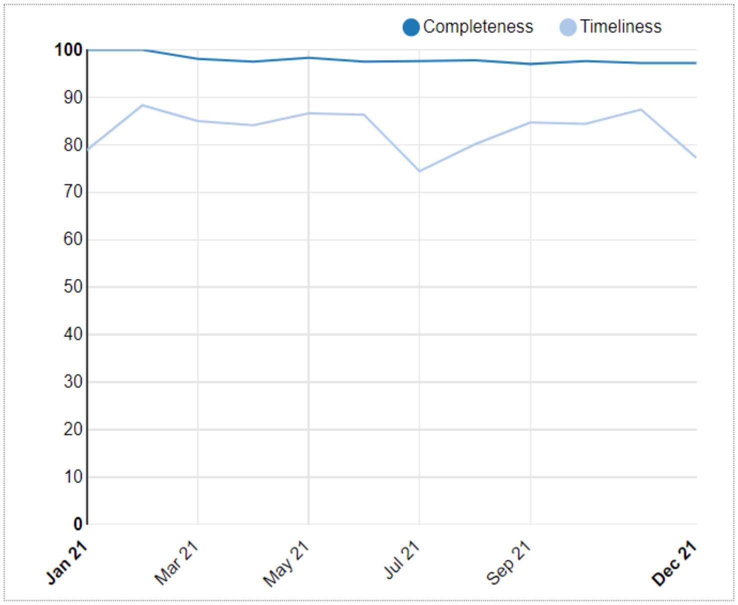

Appendix S2: Adjustment of seasonality effect

The general form of segmented regression is as follows,

$$Y_t = \beta_0 + \beta_1 * time_t + \beta_2 intervention_t + \beta_3 * time\ after\ intervention_t + e_t$$

Where, Y_t represents the mean number of deaths in month t; ***time*** is a continuous variable indicating the time in months from the start of the observation period; ***intervention*** refers to any event, such as the COVID-19 pandemic, being an indicator at time t with a value of ***0*** before the event and ***1*** after; and ***time after intervention*** is a continuous variable counting the number of months after the intervention at time t, coded ***0*** before the covid-19 pandemic and continuing from ***1*** after the covid-19 pandemic.

Seasonal patterns are modelled as an additional term that repeats every 12 months (1). Based on the Fourier series expansion theorem, any periodic signal with a time period ***T*** can be represented as a sum of sine and cosine functions. For monthly data, with ***T = 12***, we used Fourier series to fit the seasonality component, that is, linear combinations of sine and cosine functions. For example, to model monthly counts, the model can be written as follows (2),

$$Y_t = \beta_0 + \beta_1 * time_t + \beta_2 intervention_t + \beta_3 * time\ after\ intervention_t + \beta_s * sin(\frac{2\pi t}{T}) + \beta_c * cos(\frac{2\pi t}{T}) + e_t$$

References:

1. Bramness JG, Walby FA, Morken G, Røislien J. Analyzing seasonal variations in suicide with Fourier Poisson time-series regression: a registry-based study from Norway, 1969–2007. American journal of epidemiology. 2015;182(3):244-54.

2. Ramanathan K, Thenmozhi M, George S, Anandan S, Veeraraghavan B, Naumova EN, et al. Assessing seasonality variation with harmonic regression: accommodations for sharp peaks. International journal of environmental research and public health. 2020;17(4):1318.
